# Cerebellar theta burst stimulation modulates short latency afferent inhibition in Alzheimer's disease patients

**DOI:** 10.3389/fnagi.2013.00002

**Published:** 2013-02-19

**Authors:** Francesco Di Lorenzo, Alessandro Martorana, Viviana Ponzo, Sonia Bonnì, Egidio D'Angelo, Carlo Caltagirone, Giacomo Koch

**Affiliations:** ^1^Neurologia Clinica e Comportamentale, Fondazione Santa Lucia IRCCSRome, Italy; ^2^Dipartimento di Neuroscienze, Policlinico Tor Vergata, Università di Roma Tor VergataRome, Italy; ^3^Dipartimento di Sanità Pubblica e Neuroscienze, Università di Pavia, and IRCCS C. MondinoPavia, Italy

**Keywords:** transcranial magnetic stimulation, cortical plasticity, cholinergic, cerebellum, Alzheimer's disease

## Abstract

The dysfunction of cholinergic neurons is a typical hallmark in Alzheimer's disease (AD). Previous findings demonstrated that high density of cholinergic receptors is found in the thalamus and the cerebellum compared with the cerebral cortex and the hippocampus. We aimed at investigating whether activation of the cerebello-thalamo-cortical pathway by means of cerebellar theta burst stimulation (TBS) could modulate central cholinergic functions evaluated *in vivo* by using the neurophysiological determination of Short-Latency Afferent Inhibition (SLAI). We tested the SLAI circuit before and after administration of cerebellar continuous TBS (cTBS) in 12 AD patients and in 12 healthy age-matched control subjects (HS). We also investigated potential changes of intracortical circuits of the contralateral primary motor cortex (M1) by assessing short intracortical inhibition (SICI) and intracortical facilitation (ICF). SLAI was decreased in AD patients compared to HS. Cerebellar cTBS partially restored SLAI in AD patients at later inter-stimulus intervals (ISIs), but did not modify SLAI in HS. SICI and ICF did not differ in the two groups and were not modulated by cerebellar cTBS. These results demonstrate that cerebellar magnetic stimulation is likely to affect mechanisms of cortical cholinergic activity, suggesting that the cerebellum may have a direct influence on the cholinergic dysfunction in AD.

## Introduction

Alzheimer's disease (AD) pathophysiology is dominated by a dysfunction of the central cholinergic system. In AD patients, the impairment of central cholinergic functions can be evaluated *in vivo* by using a neurophysiological effect called Short-Latency Afferent Inhibition (SLAI) (Tokimura et al., 2000). SLAI consists in the inhibition of the Motor Evoked Potentials (MEPs) by afferent sensory impulses. SLAI can be easily measured by applying an electric conditioning pulse on the median nerve at wrist that precedes the TMS test pulse applied over the contralateral primary motor cortex (M1) by 20–25 ms. SLAI is abolished by scopolamine, a potent muscarinic antagonist (Di Lazzaro et al., 2002), and it has therefore been suggested that the inhibitory effect of peripheral stimulation is mediated by cholinergic projections over the primary motor cortex. In AD patients, SLAI is reduced to various degrees depending on the severity of the disease, so that the decreased inhibitory effect of peripheral stimulation is thought to reflect the cholinergic dysfunction in AD (Di Lazzaro et al., 2002; Martorana et al., 2009).

Although the cerebellum is not among the most renown brain structures to be affected by the pathology, recent evidence suggested that it undergoes degenerative changes in AD: the posterior cerebellar lobes are significantly smaller in AD patients when compared to HC, and atrophy of the posterior cerebellar regions is associated with poorer cognitive performance (Thomann et al., 2008). Moreover, the cerebellum is strongly involved in cholinergic functions. A recent PET study demonstrated that intravenously administered [^11^C]-donepezil, an acetyl-cholinesterase (AChE) inhibitor used in AD therapy, rapidly enters the brain and mainly distributes to the striatum, thalamus, and cerebellum, which are known to contain high densities of AChE compared with the cerebral cortex and hippocampus (Okamura et al., 2008). The regional distribution of [^11^C]-donepezil was consistent with regional AChE activity determined in a human postmortem study (Finkelstein et al., 1988). Moreover, nicotinic cholinergic receptors (nAChRs) are widely distributed in the mammalian cerebellum and are known to regulate synaptic efficacy at two major classes of cerebellar neurons (Turner et al., 2011; D'Angelo and Casali, 2012).

In humans, the neural activity of the cerebellum can be explored *in vivo* by means of repetitive transcranial magnetic stimulation (Ugawa et al., 1995; Del Olmo et al., 2007; Koch et al., 2008). Therefore, in the current study, we sought to investigate whether cerebellar magnetic stimulation could modulate the altered SLAI circuits described in AD patients. We reasoned that given that the cerebellar activity is involved in the cholinergic system, cerebellar continuous TBS (cTBS) could provide novel information regarding the interactions between the cerebello-thalamo-cortical circuits and the central cholinergic functioning in AD patients.

## Materials and methods

### Subjects

We examined 12 patients with a new diagnosis of probable AD according to the NINCDS-ADRDA criteria (Varma et al., 1999) and 12 neurologically healthy age-matched control subjects (HS). The mean (±SD) age of the patients was 69.8 (±4.9) years, whereas that of controls was 71.7 (±4.4) years. All patients underwent a complete clinical investigation, including medical history, neurological examination, mini mental state examination (MMSE), a complete blood screening (including routine exams, thyroid hormones, level of B12), neuropsychological examination, a complete neuropsychiatric evaluation, and morphological magnetic resonance imaging (1.5 T MRI). Exclusion criteria were the following: patients with isolated deficits and/or unmodified MMSE (≥25/30) on revisit (6, 12, and 18 months follow-up), patients with clinically manifest acute stroke in the last 6 months showing Hachinsky scale score >4, and a radiological evidence of sub-cortical lesions. None of patients revealed pyramidal and/or extrapyramidal signs at the neurological examination. At the time of enrolment, in the 30 days before participating in this study, none of the patients had been treated with drugs that might have modulated cerebral cortex excitability such as antidepressants, or any other neuroactive drugs (i.e., benzodiazepines, anti-epileptic drugs, or neuroleptics), and they had not been treated with cholinesterase inhibitors. All AD patients showed a cognitive profile consistent with moderate dementia, as assessed by a neuropsychological evaluation including the MMSE and a standardized neuropsychological battery (Carlesimo et al., 1996). On the MMSE, AD patients scored a mean of 21.08 (±3.9) and Clinical Dementia Rating (CDR) was 1.21 (±1.1). All participants or their legal guardian gave the written informed consent after receiving an extensive disclosure of study. The study was performed according to the Declaration of Helsinki and approved by the ethics committee of the Tor Vergata University in Rome.

### Experiment 1: effects of cerebellar cTBS on SLAI, SICI, and ICF circuits

#### SLAI

Magnetic stimulation was performed using an high power Magstim 200 magnetic stimulator (Magstim Co, Whitland, Dyfed, UK). The magnetic stimuli had a nearly monophasic pulse configuration, with a rise time of 0.1 ms, decaying back to zero over 0.8 ms. A figure of eight coil with external loop diameters of 9 cm was held over the left motor cortex at the optimum scalp position to elicit motor responses in the contralateral first dorsal interosseous (FDI) muscle. The optimal position was marked on the scalp with a felt pen to ensure identical placement of the coil throughout the experiment. The handle of the coil pointed backward and was perpendicular to the presumed direction of the central sulcus, about 45° to the midsagittal line. The direction of the induced current was from posterior to anterior and was optimal to activate the motor cortex trans-synaptically. Surface muscle responses were recorded via two 9 mm diameter Ag–AgCl electrodes with the active electrode over the motor point of the muscle and the reference on the metacarpophalangeal joint of the index finger. Muscle responses were amplified and filtered (bandwidth 3–3000 Hz) by D150 amplifiers (Digitimer, Welwyn Garden City, Hertfordshire, UK). Data were collected on a computer with a sampling rate of 10 kHz per channel and stored for later analysis using a CED 1401 A–D converter (Cambridge Electronic Design, Cambridge, UK). All the AD patients selected were able to understand and carry out the simple task required for this electrophysiological study—that is, to keep fully relaxed.

**Figure 1:**
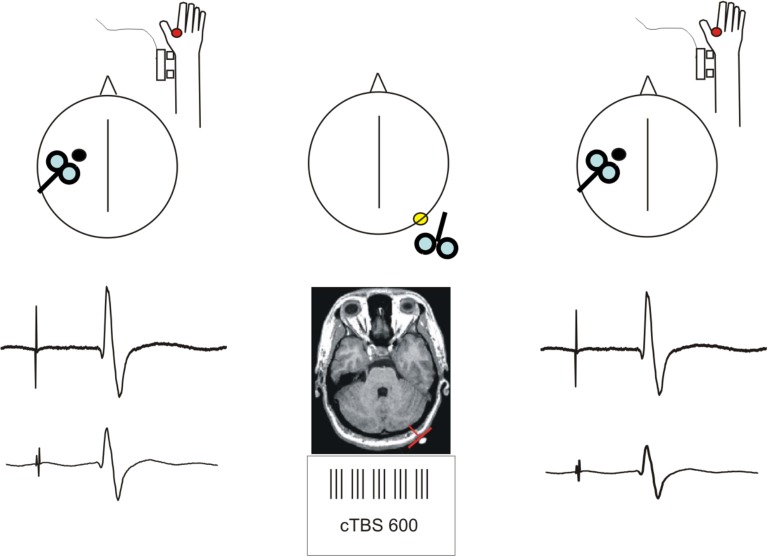
**Schematic description of the experimental procedure**. Short-latency afferent inhibition (SLAI) was recorded by measuring the inhibitory effects of the peripheral median nerve stimulation over the amplitude of the MEP recorded from the right FDI at different interstimulus intervals in AD patients and age matched healthy controls. SLAI was measured at baseline and after a session of cTBS over the left lateral cerebellum in both groups.

The resting motor threshold (RMT) was defined as the lowest intensity that produced MEPs of >50 μ V in at least five out of 10 trials with the muscles relaxed (Rossini et al., 1994). Determination of RMT was done in step width of 1% of maximal stimulator output (MSO). Short latency inhibition was studied using the technique that has been recently described (Tokimura et al., 2000) (see Figure 1). Conditioning stimuli were single pulses (200 μs) of electrical stimulation applied through bipolar electrodes to the right median nerve at the wrist (cathode proximal). The intensity of the conditioning stimulus was set at just over motor threshold for evoking a visible twitch of the thenar muscles. The intensity of the test cortical magnetic stimulus was adjusted to evoke a muscle response in relaxed right FDI with amplitude of approximately 1 mV peak to peak. The conditioning stimulus to the peripheral nerve preceded the magnetic test stimulus by different interstimulus intervals (ISIs). ISIs were determined relative to the latency of the N20 component of the somatosensory evoked potential induced by stimulation of the right median nerve. The active electrode for recording the N20 potential was attached 3 cm posterior to C3 (10–20 system) and the reference was 3 cm posterior to C4. Five hundred responses were averaged to identify the latency of the N20 peak. ISIs from N20 −4 ms to N20 +8 ms were investigated in 4 ms steps. Ten stimuli were delivered at each ISI. The subject was given audiovisual feedback at high gain to assist in maintaining complete relaxation. The inter-trial interval was set at 5 s (±10%), for a total duration of approximately 5 min. Measurements were made on each individual trial. The mean peak-to peak amplitude of the conditioned MEP at each ISI was expressed as a percentage of the mean peak-to-peak amplitude size of the unconditioned test pulse in that block.

#### SICI-ICF

We used a 7 cm figure-of-eight coil connected with two Magstim 200 stimulators to apply paired TMS over the motor cortex. In order to investigate M1 intracortical circuits such as short intracortical inhibition (SICI) and intracortical facilitation (ICF). The magnetic stimuli had a nearly monophasic pulse configuration. The coil was placed at the optimal position for eliciting MEPs from the left contralateral FDI muscle. SICI and ICF were tested using paired TMS with a conditioning stimulus (CS) preceding a test stimulus (TS) by 1–15 ms (Kujirai et al., 1993; Ziemann et al., 1996). CS was set at 80% AMT (Huang and Rothwell, 2004) while the intensity of TS was adjusted to evoke a MEP of approximately 1 mV peak to peak in the relaxed FDI. The amplitude of the conditioned MEP at each ISI was expressed as a percentage of the mean peak-to-peak amplitude size of the unconditioned TS in that block.

#### cTBS

A MagStim Super Rapid magnetic stimulator (Magstim Company, Whitland, Wales, UK), connected with a figure-of-eight coil with a diameter of 70 mm was used to deliver cTBS. Three-pulse bursts at 50 Hz repeated every 200 ms for 40 s were delivered at 80% of the active motor threshold (AMT) over the lateral cerebellum (600 pulses) (Huang et al., 2005). cTBS was applied over the right lateral cerebellum using the same scalp co-ordinates (1 cm inferior and 3 cm left/right to the inion) adopted in previous MRI studies showing that this site target the posterior and superior lobules of the lateral cerebellum (Del Olmo et al., 2007). We used the figure-of-eight coil, since this approach has been adopted in previous investigations in which cerebellar rTMS was shown to be effective in modulating the excitability of the contralateral motor cortex (Del Olmo et al., 2007). The coil was positioned tangentially to the scalp, with the handle pointing superiorly.

SLAI and SICI/ICF were tested before in two different blocks and immediately after the application of cTBS over the right lateral cerebellum. The order of presentation of the blocks before and after cTBS was pseudo-randomized across subjects.

### Experiment 2: effects of cerebellar cTBS on SLAI input-output curves in healthy subjects

SLAI is already different between groups (less in AD) at baseline. Therefore, it is unclear how to interpret the effects of cerebellar cTBS. From the reported data, the possibility cannot be excluded that the modulating cTBS effect was not seen in the healthy controls merely due to SLAI saturation (floor effect). To disentangle this, we performed an experiment in which we studied SLAI input-output curves by systematic variation of the intensity of the peripheral nerve stimulus to compare the effects of cTBS at equivalent SLAI levels as those obtained in the AD patients group. In a group of eight healthy controls SLAI was tested as in Experiment 1, but three different blocks were applied. In each block the intensity of the peripheral nerve stimulation was set at 100, 200, and 300% of the sensory threshold (ST) (note that an intensity of 300% is close to the one necessary for evoking a visible twitch of the thenar muscles as in Experiment 1). SLAI blocks were tested before and immediately after the application of cTBS over the right lateral cerebellum. The order of presentation of the blocks before and after cTBS was pseudo-randomized across subjects.

### Experiment 3: effects of occipital cTBS on SLAI, SICI, and ICF circuits

We performed an additional experiment in order to exclude that the cTBS effects obtained in the AD patients could be non-specific. Therefore, we performed the same experiment as in Experiment 1 but we varied the site of application of cTBS, by choosing the occipital cortex as a control area. The occipital TMS site was 3 cm above the inion and 1 cm right of midline (Romei et al., 2012). SLAI and SICI/ICF were tested before in two different blocks and immediately after the application of cTBS over the right lateral cerebellum. The order of presentation of the blocks before and after cTBS was pseudo-randomized across subjects.

### Statistical analysis

In Experiment 1 SLAI parameters of AD patients were compared with those of controls by means of repeated measures ANOVA with GROUP (AD vs. healthy subjects) as between subjects factor and ISI (−4, 0, +4, and +8 ms plus the latency of the N20) and PROTOCOL (pre vs. post cTBS) as within subjects factors. For SICI we performed a repeated measures analysis ANOVA with GROUP (AD vs. healthy subjects) as between subjects' factor and ISI (1, 2, 3, 5, 7, 10, and 15 ms) and PROTOCOL (pre vs. post cTBS) as within subjects factors. In experiment 2 we performed a repeated measures ANOVA with INTENSITY (100, 200, and 300% of ST), ISI (−4, 0, +4, and +8 ms plus the latency of the N20) and PROTOCOL (pre vs. post cTBS) as within subjects factors. In Experiment 3 SLAI parameters of AD patients were analysed by means of repeated measures ANOVA with ISI (−4, 0, +4, and +8 ms plus the latency of the N20) and PROTOCOL (pre vs. post cTBS) as within subjects factors. When a significant main effect was reached, paired *t*-tests with Bonferroni correction were employed to characterize the different effects of the specific ISIs. For all statistical analyses, a *p* value of <0.05 was considered to be significant. Mauchley's test examined for sphericity. The Greenhouse–Geisser correction was used for non-spherical data.

## Results

### Experiment 1

**Figure 2:**
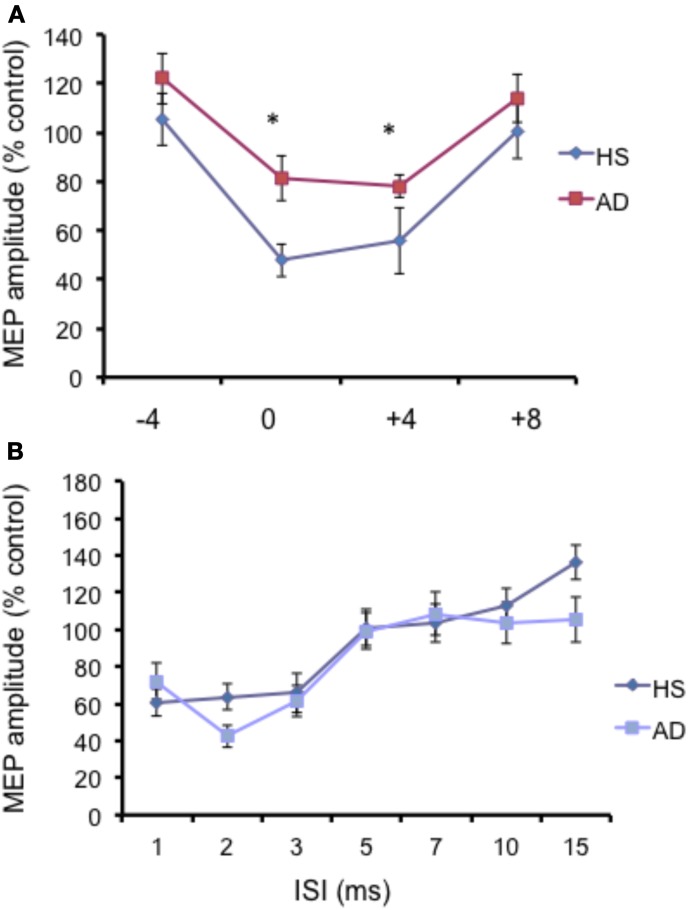
**(A)** Short-latency afferent inhibition (SLAI) at different interstimulus intervals in AD patients (open squares) and control (black diamonds) subjects. The interval between median nerve stimulation and cortical stimuli was corrected for the latency of the N20 component of the somatosensory evoked potential in each subject (see text). The size of MEPs is expressed as a percentage of the MEP evoked by magnetic stimulation alone. **(B)** Short intracortical inhibition (SICI) and intracortical facilitation (ICF) at different interstimulus intervals in AD patients (open squares) and control subjects (black diamonds). The size of MEPs is expressed as a percentage of the MEP evoked by magnetic stimulation alone. Error bars indicate mean standard errors. ^*^p < 0.05.

**Figure 3:**
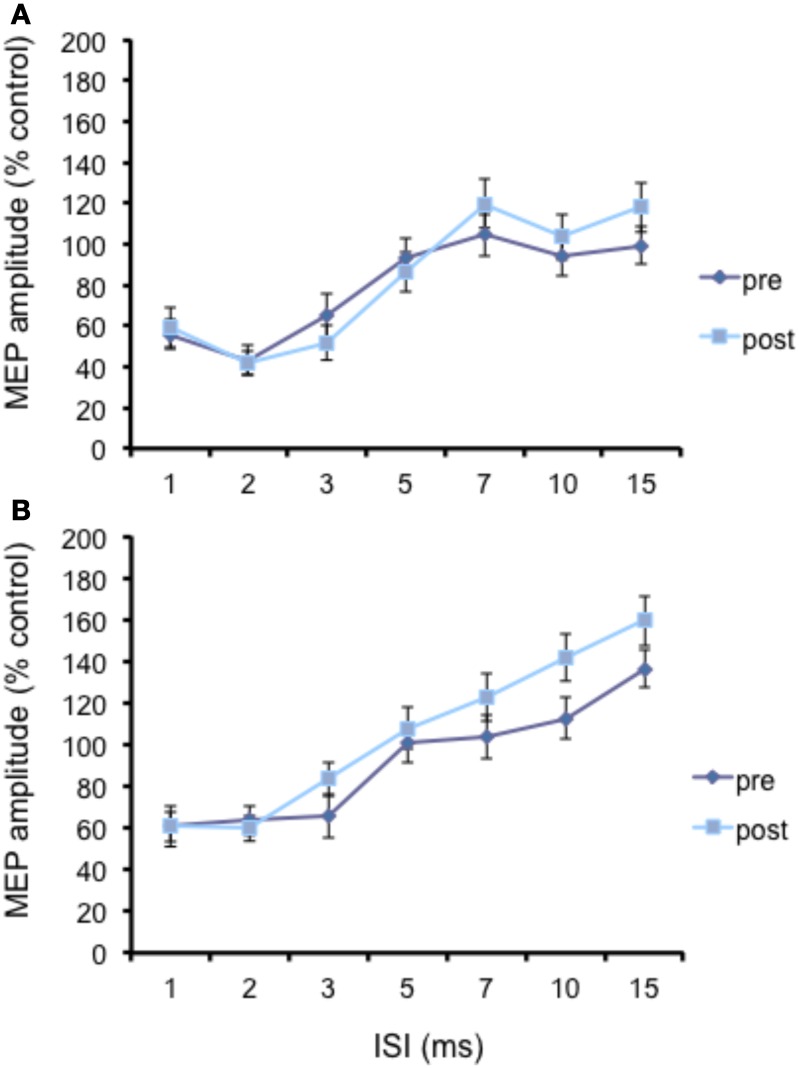
**Effects of cerebellar cTBS on SLAI (A) and SICI (B) in AD patients**. Error bars indicate standard deviations. The size of MEPs is expressed as a percentage of the MEP evoked by magnetic stimulation alone. Error bars indicate mean standard errors.

**Figure 4:**
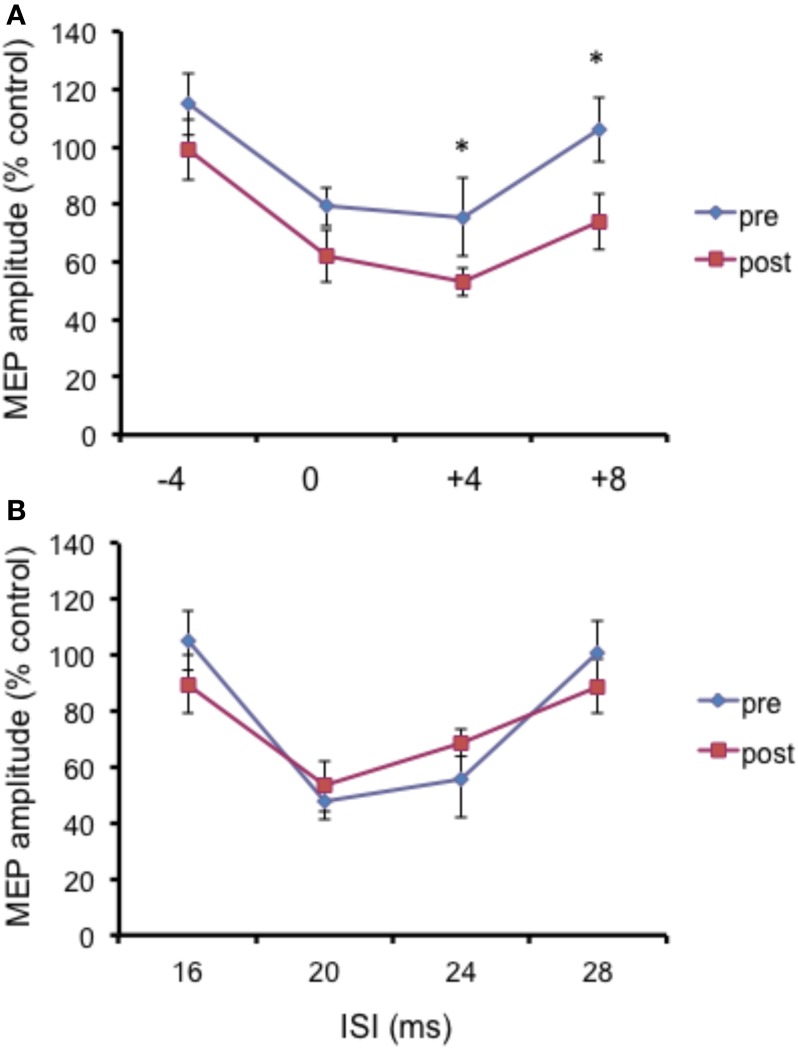
**Effects of cerebellar cTBS on SLAI (A) and SICI and ICF (B) in healthy subjects**. Error bars indicate standard deviations. The size of MEPs is expressed as a percentage of the MEP evoked by magnetic stimulation alone. Error bars indicate mean standard errors. ^*^p < 0.05.

The N20 latency and amplitude were within normal limits in all AD patients and control subjects and did not differ between the two groups (20.5 ± 3.2 ms vs. 20.8 ± 2.9 ms). The mean (SD) RMT to TMS was significantly lower in AD patients than in controls (45.9% ± 2.1 vs. 49.9 ± 1.1%) of MSO; *t* = 3.14; *p* < 0.05 (see Table 1). RMT was not significantly modified following cerebellar cTBS (*p* = 0.48 at paired *t*-test analysis). For unconditioned, TS MEPs amplitude pre and post cTBS did not differ being, respectively, 1.08 ± 0.33 mV and 1.04 ± 0.31 mV in AD and 1.13 ± 0.25 mV and 1.14 ± 0.34 mV in controls. Repeated ANOVA performed on SLAI measures revealed significant main effects of PROTOCOL (*F* = 5.66; *p* = 0.027) and ISI (*F* = 19.14; *p* = 0.0001), as well significant GROUP × PROTOCOL (*F* = 4.26; *p* = 0.041) interaction. The triple interaction GROUP × ISI × PROTOCOL was not significant. *Post hoc* analysis revealed that at baseline the amount of SLAI inhibition was smaller in AD patients than in normal controls at +4 and +8 ms (all *p* < 0.05) (Figure 2). The amount of SLAI inhibition increased in AD patients following cTBS at 0 and +4 ms ISIs (all *p* < 0.05) (Figure 3A). On the other hand when the same analysis was performed in the HS group, no significant difference emerged (Figure 3B). No effects were found for SICI and ICF in both groups. (Figures 4A,B).

**Table 1:** **Parameters of corticospinal excitability before and after cTBS**.

**Measures**	**AD**	**HS**
rMT (%)	45.9 ± 2.1	49.9 ± 1.1
aMT (%)	34 ± 1.3	37 ± 1.9
1mV (%)	52.41 ± 2.8	55.4 ± 2.2
**PRE cTBS**		
ICI 2 ms (%)	42.85 ± 7.2	65 ± 10.6
ICF 15 ms (%)	99.22 ± 9.0	136.9 ± 9.1
SLAI 24 ms (%)	75.4 ± 13.5	57.7 ± 9.8
**POST cTBS**		
ICI 2 ms (%)	41.77 ± 6.1	60.32 ± 9.6
ICF 15 ms (%)	118 ± 12.1	136.5 ± 27
SLAI 24 ms (%)	53.147 ± 4.9	63.4 ± 8.8

*rMT, resting motor threshold; aMT, active motor threshold; 1mV, intensity of magnetic stimulus adjusted to evoke 1 mV peak to peak MEP; ICI, intracortical inhibition; SLAI, short-latency afferent inhibition. For the threshold values, “%” are related to the maximal stimulator output (MSO); for SICI, ICF and SLAI values, “%” are related to the control MEP amplitude*.

### Experiment 2

**Figure 5:**
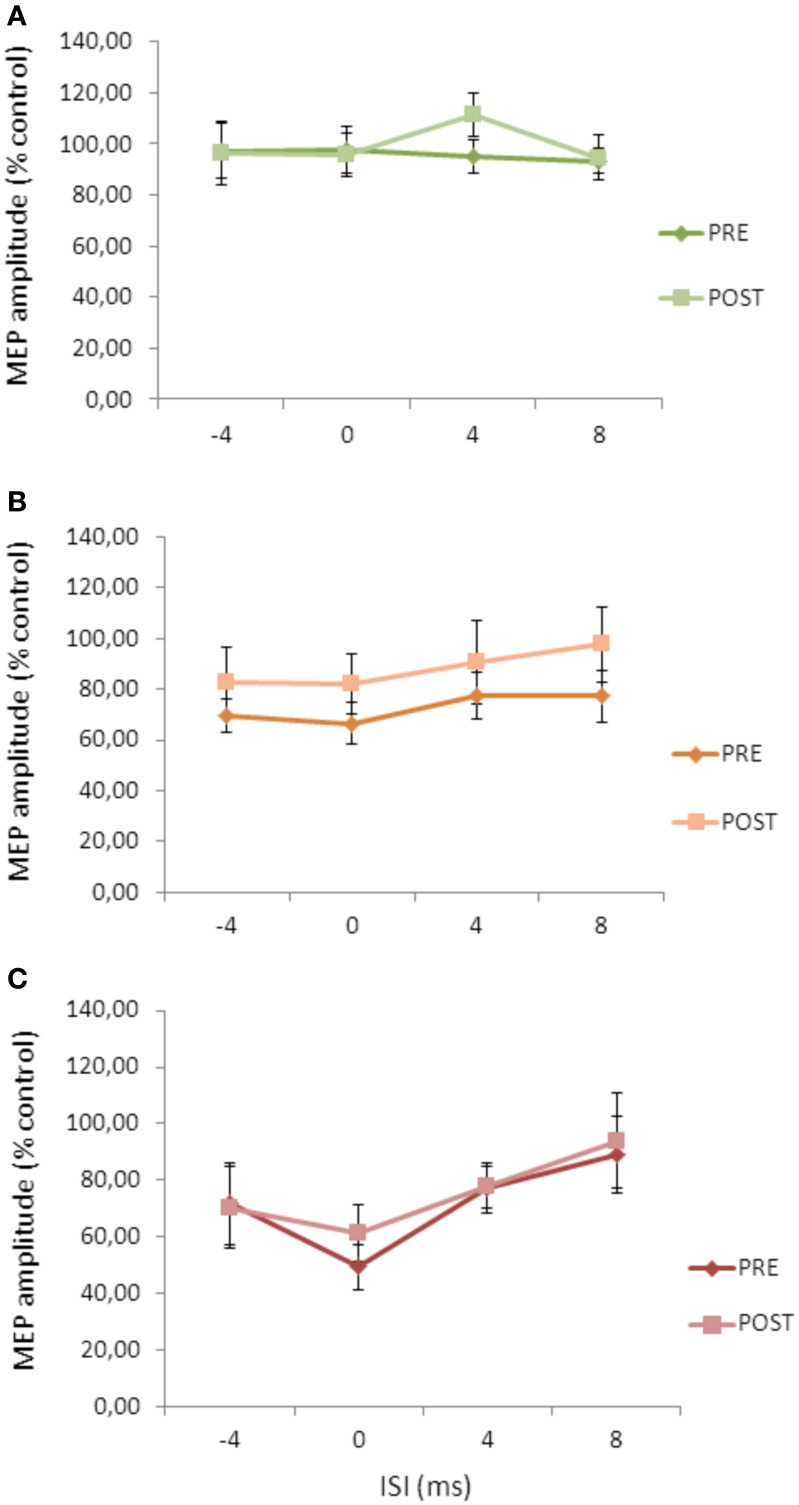
**Effects of cerebellar cTBS on SLAI applied with different intensities of peripheral nerve stimulation in healthy subjects (A) = 100%; (B) = 200%; (C) = 300% of the sensory threshold**. Error bars indicate standard deviations. The size of MEPs is expressed as a percentage of the MEP evoked by magnetic stimulation alone. Error bars indicate mean standard errors.

Repeated ANOVA performed on SLAI measures revealed significant main effects of INTENSITY (*F* = 2.14; *p* = 0.036) and ISI (*F* = 2.64; *p* = 0.04), but no a significant cTBS effect (Figure 5). All the interactions were not significant. These data indicate that the cTBS effects observed in Experiment 1 were specific for the AD patients and did not depend on a different basal level of SLAI.

### Experiment 3

**Figure 6:**
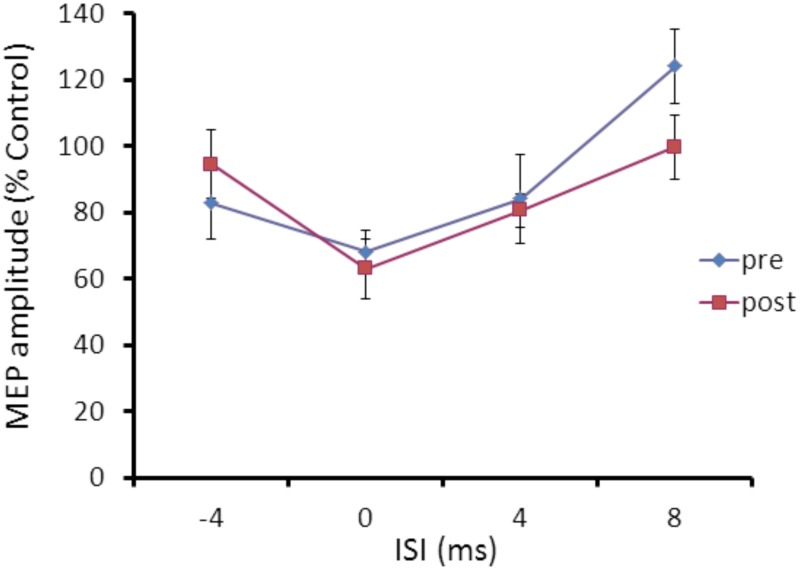
**Effects of occipital cTBS on SLAI in AD patients**. Error bars indicate standard deviations. The size of MEPs is expressed as a percentage of the MEP evoked by magnetic stimulation alone. Error bars indicate mean standard errors.

Repeated ANOVA performed on SLAI measures revealed a significant main effect of ISI (*F* = 4.04; *p* = 0.023), but no effect for cTBS main factor and for the cTBS × ISI interaction (Figure 6).

## Discussion

The current results confirmed previous studies showing that SLAI of the motor cortex is significantly reduced in AD patients compared to age matched normal subjects (i.e., Di Lazzaro et al., 2002; Freitas et al., 2011). Notably, we found that such SLAI dis-inhibition is restored following a single session of cerebellar cTBS. Our results therefore seem to suggest that the activation of cerebello-thalamo-cortical pathway by means of cerebellar cTBS is able to modulate SLAI function in AD patients. Although SLAI is considered a hallmark of central cholinergic function that is thought to depend on the integrity of cortico-cortical inhibitory circuits (Tokimura et al., 2000), its current interpretation is still debated. Acetylcholine is thought to be involved in modulation of intracortical circuits mediated by groups of GABAergic interneurons, rather than directly acting on pyramidal cells (Di Lazzaro et al., 2002, 2004). At this regard, Di Lazzaro and co-workers (Di Lazzaro et al., 2005) demonstrated that different types of benzodiazepines such as diazepam and lorazepam resulted in distinct effects on both SICI and SLAI. In particular diazepam increased both SLAI and SICI, while lorazepam reduced SLAI but increased SICI, suggesting that the interactions between cholinergic and GABAergic circuits may require the activation of a variety of different receptor subtypes. Moreover we recently showed that dopamine also plays a critical role in modulating cortical cholinergic activity, presumably interacting with such GABAergic intracortical circuits (Martorana et al., 2009). SLAI starts only a few milliseconds after the arrival of the somatosensory input at the cortex, and implies a relatively direct pathway from sensory input to motor output (Tokimura et al., 2000). The dorsomedial nucleus and intralaminar nuclei (thalamic paramedian structures) are under control of excitatory projections from pontomesencephalic cholinergic neurons (peduncolopontine and laterodorsal tegmental cholinergic nuclei) through muscarinic receptors (Jones, 2003; Steriade, 2004). These pontomesencephalic cholinergic neurons have no direct projection to the cortex but they do have a prominent indirect effect on the functional state (and excitability) of cortical neurons as they activate thalamocortical neurons (Steriade, 2004). Consistently with these premises, Oliviero et al. (2005) reported that, after a thalamic stroke that had destroyed the dorsomedian and intralaminar nuclei, a patient showed a selective attenuation of the SLAI in the ipsilesional M1.

Moreover, cerebellar cTBS is known to modulate the activation of cerebello-thalamo-cortical circuits (Koch et al., 2008, 2009). A recent PET study performed in order to detect the effects induced by cerebellar cTBS revealed that metabolic changes occur not only in the cerebellar cortex below the stimulated portion of the cortex but also in the deep cerebellar nuclei reflecting the activation of a pathway connecting these regions and likely projecting to the thalamus (Brusa et al., 2012).

Therefore, one possibility is that cerebellar cTBS could have increased SLAI efficacy by modulating the gating of the afferent input at the level of the thalamic nuclei. In fact, the physiology of the cerebellar-thalamo-cortical pathway activated by magnetic stimulation has been recently clarified. It has been proposed that cerebellar TMS activates the Purkinje cells of the superior cerebellum; such activation results in an inhibition of the dentate nucleus, which is known to exert a background tonic facilitatory drive onto the contralateral motor cortex (M1) through synaptic relay in the ventral lateral thalamus (Dum and Strick, 2003). This in turn leads to an inhibition of the contralateral M1, due to a reduction in dentato-thalamo-cortical facilitatory drive (Ugawa et al., 1994, 1997; Pinto and Chen, 2001; Daskalakis et al., 2004). Although apparently SLAI and the cerebello-thalamo-cortical pathway activated by cerebellar TMS act on different thalamic nuclei, it has to be considered that intrathalamic connections have been recently described between thalamic nuclei such as the ventroposterior and the medial posterior nucleus (Crabtree et al., 1998). At this regard, one could argue that the thalamus could be a plausible site for the interaction occurring between these two pathways. In alternative, it is possible that the effects that we observe could depend on an interaction occurring at the level of the primary motor cortex. Here, complex interactions of intracortical circuits that mediate both the afferent volleys from the thalamus could be responsible for the observed effects induced by cerebellar cTBS on SLAI in AD patients.

Another final possibility is that the observed results could depend on the modulation of cerebellar activity itself, not involving necessarily interconnected pathways. Notably, the effects of cerebellar cTBS were evident at later delays (ISIs = +4 and +8 ms). It has been proposed that sensory signals to the motor cortex arriving at later delays around 25 ms (corresponding approximately to the ISI = 4 ms in the current study) are transmitted by a longer polysynaptic pathway which includes the cerebellum. This view is supported by the literature showing that the cerebellum receives sensory information (Dean et al., 2010), and that patients with cerebellar degeneration have abnormal sensory-motor integration (Tamburin et al., 2003). Accordingly, recent studies performed in healthy subjects showed that both transcranial direct stimulation (TDCS) and cTBS applied over the cerebellum interfered with the long lasting effects induced by protocols of paired associative stimulation (PAS) using an electrical stimulus to the median nerve with a TMS pulse given 25 ms later to the motor cortex (Hamada et al., 2012a,b; Popa et al., 2013). This would explain why in the current study the interaction of cerebellar stimulation with sensory-motor integration is evident only for those stimuli that have longer delays. At this regard, a large limitation of the current study is that we did not investigate to which extent the observed SLAI modulation was eventually associated by changes in cerebello-thalamo-cortical inhibition (CBI). Thus, it remains to be clarified if there are any physiological relationships among CBI and SLAI circuits in AD patients. Moreover we did not even assess changes in MEP sizes after cerebellar cTBS because our main aim was to study modulation of cholinergic circuits. It has to be acknowledged that this lack of systematic investigation of test MEP sizes as well as conditioning stimulus intensities for SAI, SICI, and ICF limits the scientific quality of this paper.

It is important to notice that we did not find the expected modulation of some intracortical circuits such as SICI in the primary motor cortex that we previously described following cerebellar theta burst stimulation (TBS) (Koch et al., 2008). This could possibly depend on the older age of the healthy controls. Further experiments would be necessary to better clarify the interplay between cerebellar cTBS and cortical excitability. Moreover, we did not find any difference for SICI and ICF measurements at baseline between the two groups. This is consistent with the data presented in a recent review that considers all the studies evaluating SICI in AD (Freitas et al., 2011): some reductions of SICI to paired-pulse TMS were found by some investigators, but most (7 of 11) studies did not find differences in SICI between AD patients and controls.

Indeed, the effects of cerebellar cTBS were evident only in AD patients and not in healthy age matched controls. It could well be that we detected any change in the healthy controls because SLAI inhibition reached a floor level or, in alternative that such modulation would occur only when a deficient cholinergic innervation coexists such as in AD patients.

As expected, we found lower RMT in the AD patients group. It is therefore likely that the I-wave component of the test MEP sizes (Hamada et al., 2012a,b) would be different between AD patients and healthy controls. This different amount of I-waves could have been involved in determining the amount of SLAI and hence affected the present results. Besides, the conditioning stimulus intensity of SICI and ICF would also be different between the two groups, and therefore this could be another potential confounding factor.

Whatever the neurophysiological mechanisms underlying these complex interactions, the current data suggest that cerebellar magnetic stimulation could be effective in modulating central cholinergic activity in AD patients. Further studies aimed to investigate systematically the impact of this protocol on different cognitive functions would be important to further understand the potential clinical importance of the current findings.

### Conflict of interest statement

The authors declare that the research was conducted in the absence of any commercial or financial relationships that could be construed as a potential conflict of interest.
